# Heme Oxygenase-1 Regulates Matrix Metalloproteinase MMP-1 Secretion and Chondrocyte Cell Death via Nox4 NADPH Oxidase Activity in Chondrocytes

**DOI:** 10.1371/journal.pone.0066478

**Published:** 2013-06-20

**Authors:** Francis Rousset, Minh Vu Chuong Nguyen, Laurent Grange, Françoise Morel, Bernard Lardy

**Affiliations:** 1 Université Joseph Fourier, AGIM GREPI FRE CNRS 3405, Grenoble, France; 2 Rheumatology Department, Grenoble Teaching Hospital, CHU Hôpital Sud, Grenoble, France; 3 Laboratoire de Biochimie Enzyme et Protéine-DBTP, Institut de Biologie et de Pathologie, Centre Hospitalier Universitaire (CHU), Grenoble, France; INRS, Canada

## Abstract

Interleukin-1β (IL-1β) activates the production of reactive oxygen species (ROS) and secretion of MMPs as well as chondrocyte apoptosis. Those events lead to matrix breakdown and are key features of osteoarthritis (OA). We confirmed that in human C-20/A4 chondrocytes the NADPH oxidase Nox4 is the main source of ROS upon IL-1β stimulation. Since heme molecules are essential for the NADPH oxidase maturation and activity, we therefore investigated the consequences of the modulation of Heme oxygenase-1 (HO-1), the limiting enzyme in heme catabolism, on the IL-1β signaling pathway and more specifically on Nox4 activity. Induction of HO-1 expression decreased dramatically Nox4 activity in C-20/A4 and HEK293 T-REx™ Nox4 cell lines. Unexpectedly, this decrease was not accompanied by any change in the expression, the subcellular localization or the maturation of Nox4. In fact, the inhibition of the heme synthesis by succinylacetone rather than heme catabolism by HO-1, led to a confinement of the Nox4/p22^phox^ heterodimer in the endoplasmic reticulum with an absence of redox differential spectrum highlighting an incomplete maturation. Therefore, the downregulation of Nox4 activity by HO-1 induction appeared to be mediated by carbon monoxide (CO) generated from the heme degradation process. Interestingly, either HO-1 or CO caused a significant decrease in the expression of MMP-1 and DNA fragmentation of chondrocytes stimulated by IL-1β. These results all together suggest that a modulation of Nox4 activity via heme oxygenase-1 may represent a promising therapeutic tool in osteoarthritis.

## Introduction

The pathogenesis of osteoarthritis involves an imbalance between anabolic and catabolic pathways in chondrocytes. The expression of matrix metalloproteinases (MMPs), chondrocyte hypertrophy and apoptosis are the main features of the pathology. Proinflammatory cytokines, such as interleukin-1β (IL-1β) and tumor necrosis factor-α (TNF-α) cause damages to cartilage via the synthesis and the secretion of MMPs, which in turn lead to matrix degradation [1], [2], [3], [4], [5]. Indeed, elevated levels of IL-1β were found in OA synovial fluid, and the expression of IL-1β gene is up-regulated in OA cartilage [6]. In response to IL-1β, articular chondrocytes actively produce reactive oxygen species (ROS). ROS have been suggested to act as secondary messengers in bovine chondrocytes and are involved in AP-1 and NF-kappaB activation pathways leading to the transcription of cytokine-induced MMP-1 and MMP-13 metalloproteinases [7], [8], [9], [10]. The maturity-arrested differentiation state, also called hypertrophic differentiation that could promote OA progression, is observed among OA chondrocytes *in situ*. Indeed, hypertrophic chondrocytes undergo apoptosis leading to a dysregulation of matrix repair mechanism [11]. The role of ROS is nowadays well established in this terminal differentiation [12], [13], [14].

ROS are important regulators of redox-sensitive cell-signalling pathways and are involved in various biological processes including host defence, cell signalling, oxygen sensing, cell proliferation, differentiation and apoptosis. However, overproduction of ROS has been associated with many pathological conditions such as inflammation, vascular atherosclerosis, diabetes, hypertension, tumorigenesis, neurodegenerative pathologies and osteoarthritis [15], [16]. NADPH oxidases (Nox) function of is solely dedicated to the production of ROS. Nox2 NADPH oxidase activity in phagocyte is well characterized and plays an essential role in innate immunity [17]. Nox family is composed of seven members who share specific structural homology regions [18]. Among them, Nox4 represents the major source of ROS in kidney and is widely expressed in many others tissues including the vascular system and bones [19], [20]. For example, in osteoclasts, ROS production by Nox4 has been shown to directly contribute to bone resorption [20].

Despite evidences of ROS implication in OA, only few studies investigated the function of NADPH oxidase in human chondrocytes. Recently, Coustry and co-workers demonstrated that Nox4 mRNA is 100 fold upregulated in a chondrocyte model of pseudoachondroplasia characterized by abnormal joint architecture, joint erosion and osteoarthritis [21]. Our previous data showed that the oxidase complex Nox4/p22^phox^ is expressed in the C-20/A4 human chondrocyte cell line and represents an important source of ROS generation [22]. In this cell line, Nox4 activity modulates MMP-1 expression and DNA fragmentation upon IL-1β treatment, events that are implicated in OA pathogenesis. Consequently, targeting Nox4 activity may represent a promising therapeutic strategy against osteoarthritis.

NADPH oxidase catalytic activity is dependent on two heme molecules which transfer electrons from the FAD to molecular oxygen to give rise to superoxide anion (O_2_°^−^). Heme oxygenase-1 (HO-1) is the limiting enzyme in heme catabolism. HO-1 catalyzes the linearization of the heme molecule to produce an equimolar amount of biliverdine (BV) and carbon monoxide (CO). BV is subsequently converted to bilirubin (BR) by the NADPH biliverdin reductase. BV and BR are potent antioxidants [23] while CO has strong anti-inflammatory properties [24]. During Nox2 maturation, heme is incorporated into a 65 kDa precursor allowing its dimerization with the stabilizing subunit p22^phox^ [25]. The complex called cytochrome *b*_558_ (cyt *b*_558_) is then glycosylated and addressed to the plasma membrane. In presence of succinylacetone, a heme synthesis inhibitor, the maturation process was markedly reduced causing a significant decrease of NADPH oxidase activity [26].

In this study, we investigate whether the modulation of HO-1 expression could inhibit Nox4 activity and consequently reduce MMP-1 secretion and cell death. We first observed that the inhibition of heme synthesis by succinylacetone affected profoundly Nox4/p22^phox^ heterodimer formation and subcellular localization emphasising the role of heme synthesis in the Nox4/p22^phox^ maturation. Conversely, heme catabolism by HO-1 decreased Nox4 activity in the C-20/A4 chondrocytes and in the HEK293 T-REx™ Nox4 cells without affecting the Nox4/p22^phox^ heterodimer formation and plasma membrane addressing. In fact, our results suggest that HO-1 inhibits Nox4 activity through carbon monoxide generated through the heme degradation process. Interestingly, in chondrocyte stimulated by IL-1β, inhibition of Nox4 activity by HO-1 led to a significant decrease in the expression of the MMP-1, DNA fragmentation and cell death. This suggests that the cartilage homeostasis provided by chondrocytes could depend on the balance between Nox4 and HO-1 expression, and may qualify Nox4 and HO-1 as potential therapeutic targets in osteoarthritis.

## Materials and Methods

### Material

Chemical reagents used in this study and their sources were the following: Dulbecco's modified Eagle's medium (DMEM), fetal bovine serum (FBS) and geneticin (G418) (life technologies, Saint Aubin, France); blasticidin (Funakoshi Co, Tokyo, Japan); AMV Reverse transcriptase (QBiogene, Illkirch, France); Zero Blunt TOPO PCR cloning kit, TRIzol® reagent, Taq polymerase, Alexa Fluor 488, 546 or 633 labelled goat anti-mouse IgG, Alexa Fluor 546 anti-rabbit, Alexa Fluor 546 anti-goat, Hoechst 33258, pEFb and pcDNA3.1 plasmids (Invitrogen, Cergy Pontoise, France); ECL Western Blotting detection reagents and Goat anti-Mouse IgG-HRP antibody (GE healthcare, Buckingamshire, England); Na_4_P_2_O_7_, Na_3_VO_4_, PMSF, luminol, Horseradish Peroxidase (HRPO), Triton X-100, protoporphyrin-IX cobalt chloride (CoPP-IX), succinylacetone (SA), tricarbonylchlororutenium (II) dimer (CORM-II), ruthenium III chloride (RuCl) and bilirubin (BR) (SIGMA, Saint Quentin Fallavier, France); Biliverdin hydrochloride (BV) (tebu-bio, Le Perray en Yvelines, France); Okadaic acid, leupeptin, pepstatin, trypsin inhibitor, TLCK, human interleukin-1β, complete mini EDTA-free protease inhibitor EASYpack, FuGENE transfection reagent and lactate dehydrogenase (LDH) optimized kits (Roche, Meylan, France); diisopropylfluorophosphate (DFP, Acros Organics, Halluin, France); polyclonal Ig from goat against HO-1 (sc-7695) or against actin (sc-1615) and control Ig (Santa Cruz Biotechnologies, Heidelberg, Germany); Lab-Tek chambered coverglass (Thermo scientific, Courtaboeuf, France); anti-MMP-1 monoclonal antibody (mAb 901) (R&D Systems, Lille, France); Rabbit polyclonal Ig against calnexin (ab10286) or against VDAC (ab28777) (Abcam, Paris, France); mammalian expression plasmid encoding for Nox4A or Nox4B (pEFb) [22]; pcDNA 3.1 Nox4GFP and Nox4 monoclonal antibodies (8E9 and 6B11) [27].

### Cell Lines and Culture

Human C-20/A4 chondrocyte cell line is a gift from Dr M.B. Goldring (Harvard Institute of Medicine, Boston, MA, USA) [5]. HEK293 T-REx™ Nox4 cells were provided by Pr KH. Krause (Geneva University, PATIM laboratory, Switzerland) [28]. Cells were cultured in DMEM containing 4.5 g/L glucose and 0.11 g/L sodium pyruvate, supplemented with 10% (v/v) fetal bovine serum, 100 units/ml penicillin, 100 mg/ml streptomycin and 2 mM glutamine at 37°C in atmosphere containing 5% CO_2_ [27]. Blasticidin (5 µg/ml) and G418 (400 µg/ml) were used for HEK293 T-REx™ Nox4 cells. For experiments with IL-1β, complete cell culture medium was replaced by serum free DMEM. C-20/A4 chondrocytes were then stimulated with IL-1β supplemented or not with antioxidants during 48h for MMP-1 assay and 5 days for cell death assessment. All experiments were performed within cell passages 3 to 10 at 60–90% confluence.

### Generation of Plasmid Constructs for Heme Oxygenase-1 (HO-1) Expression

Human HO-1 cDNA was obtained by PCR after RNA extraction and cDNA amplification from CoPP-IX treated C-20/A4 chondrocytes. HO-1 sequence amplification was realized with the forward primer HO-1-F1 (5′ GTTT**AAGCTT**ATGGAGCGTCCGCAACCCGAC 3′) including a Hind III site (in bold type) and the reverse primer HO-1-R1 (5′ CTTT**CTCGAG**TCACATGGCATAAAGCCCTACAGC 3′) comprising a Xho I restriction site (in bold type). The purified HO-1 PCR product was subcloned into the pcR Blunt II-TOPO vector according to the manufacturing protocol (Zero Blunt TOPO PCR cloning kit, (Invitrogen)). pcR-Blunt II-TOPO plasmid containing HO-1 encoding sequence was digested by Hind III and Xho I, and HO-1 insert was ligated into linearized pcDNA 3.1 vector (Invitrogen). Plasmid encoding for HO-1 was checked by sequencing (Genome Express, Grenoble, France).

### Stable Transfection of Mammalian Expression Plasmids

C-20/A4 chondrocyte cells were trypsinized and counted. 4×10^5^ C-20/A4 cells were seeded in 6-well plates and allowed to grow for 24h to reach a 60% confluence in 2 mL complete DMEM culture medium. Cells were transfected with 1 µg of pEFb vectors encoding for Nox4A or Nox4B or 1 µg pcDNA3.1 vector encoding for Nox4GFP or HO-1 according to the manufacturing protocol (FuGENE, Roche). After 24 h, transfected cells were selected for 3 weeks with 10 µg/ml blasticidin for pEFb vector or 900 µg/ml geneticine for pcDNA3.1.

### Measurement of NADPH Oxidase Activity in Intact Cells by Luminescence Assay

ROS production was measured as described by Grange et al [22]. Briefly, cells were detached with trypsin, washed twice with PBS and collected by centrifugation (400 g, 8 min, RT). The viability of the suspended cells was over 90%, as determined by the trypan blue exclusion method. In a 96-well plate, 5×10^5^ living cells resuspended in 50 µl were added per well. Before the start of the assay, 200 µl of a PBS solution containing 20 µM luminol, and 10 units/ml of horseradish peroxidase was added in each well. Relative luminescence unit (RLU) counts were recorded every 30 s for a total of 45 min at 37°C using a Luminoscan® luminometer (Labsystems, Helsinki, Finland).

### Cell Extracts Preparation

Cells were treated with 3 mM DFP and lysed in Triton X-100 lysis buffer containing 20 mM Tris-HCl pH 7.4, 1% (v/v) Triton X-100, 150 mM NaCl, 1 mM EDTA, 10 mM Na_4_P_2_O_7_, 10 nM okadaic acid, 2 mM Na_3_VO_4_, 2 µg/ml leupeptin, 2 µg/ml pepstatin, 10 µg/ml trypsin inhibitor, 44 µg/ml PMSF, 10 µM TLCK and complete mini EDTA-free protease inhibitor (Triton X-100 cell extract). After 10 min incubation on ice, the mixture was centrifuged at 1000 g for 10 min at 4°C. The supernatant was then used for SDS-PAGE and Western Blotting or cytochrome *b* spectroscopy.

### Reduced Minus Oxidized Difference Spectra of Nox4

Reduced minus oxidized difference spectra were performed on Triton X-100 extract from C-20/A4 chondrocytes transfected or not with Nox4 and HMOX1 genes (HMOX1 encoded for HO-1). Reduction was achieved in the cuvette by addition of a few grains of sodium dithionite, and reduced minus oxidized difference spectra were recorded at room temperature with a DU 640 spectrophotometer (Beckman Coulter, Roissy, France). The amount of cyt *b* was determined by absorbance at 426 nm using the ε value of the cytochrome *b*_558_ of Nox2 (106 mM^−1^cm^−1^). A positive control was performed on cytochrome *b*_558_ purified from human neutrophils [29].

### SDS/PAGE and Western Blotting

Triton X-100 cell extract or centricon 10X concentrated cell culture supernatant were denatured at 60°C for 30 min or 4°C overnight and loaded on 10% or 12.5% (p/v) SDS-PAGE for migration and then electro-transfer to nitrocellulose. Immunodetection was performed using antibodies raised against Nox4 (mAb 8E9 or 6B11, 1∶500) [27], MMP-1 (1∶500), HO-1 (1∶500), Actin (1∶1000) or VDAC (1∶1000). Secondary antibody was conjugated to peroxidase (1∶5000). Peroxidase activity was detected using ECL reagents (GE Healthcare).

### Flow Cytometry

To assess the DNA fragmentation, C-20/A4 chondrocytes were fixed overnight in ice cold absolute ethanol, washed twice in PBS and 2×10^6^ cells were stained with 50 µg/ml propidium iodide in presence of 100 µg/ml RNAse. After 30 min incubation on ice, propidium iodide emission of fluorescence (615 nm) was assessed by FACS. Plasma membrane localization of Nox4 was assessed on HEK293 T-REx™ Nox4 cells induced for Nox4 expression with tetracycline and treated or not with 25 µM Cobalt Protoporphyrin-IX (CoPP-IX) or 25 µg/ml succinylacetone during 48 h. 10^6^ unpermeabilized cells were then suspended in 150 µL PBS containing 0.2% (w/v) BSA and 0.5 mM CaCl_2_ (PBS/BSA/CaCl_2_ buffer) and incubated on ice for 30 min with 5 µg of mouse monoclonal Ig (irrelevant Ig or 8E9 mAb) [27]. Cells were washed twice and resuspended in 150 µl of the Alexa Fluor 488 goat anti-mouse antibody diluted 1∶200 in PBS/BSA/CaCl_2_ buffer. After 30 min incubation on ice, cells were washed twice and fluorescence intensity was assessed. All FACS experiments were performed on a FACSCanto II cytometer (Becton Dickinson).

### Confocal Microscopy

Nox4GFP overexpressing C-20/A4 chondrocytes were seeded on coverslips for 48 h and treated or not with 25 µM Cobalt Protoporphyrin-IX or 25 µg/ml succinylacetone. When confluence reached 60%, cells were fixed with 4% (w/v) paraformaldehyde (PFA) for 10 min and PFA fluorescence was quenched by 50 mM NH_4_Cl for 10 min at RT. Nox4 subcellular localization in C-20/A4 chondrocytes was assessed by 1 h incubation at room temperature with 5 µg of mouse monoclonal Ig (irrelevant Ig or 8E9 mAb) in 200 µl of PBS containing 1% (w/v) BSA (PBS/BSA buffer). Cells were then washed twice with PBS/BSA buffer and permeabilized with 0.1% (v/v) Triton X-100 during 10 min at RT. Non treated and HO-1 induced chondrocytes were stained for 1h at RT with a goat anti HO-1 antibody (1/50 in PBS/BSA buffer). Meanwhile, succinylacetone treated cells were incubated at RT with 5 µg rabbit anti calnexin antibody in 200 µl PBS/BSA buffer. After two washes with PBS/BSA buffer, 1 h incubation with anti-goat (for HO-1 labeled cells) or anti-rabbit (for calnexin) secondary antibodies conjugated to Alexa 546 (1/200) was performed at RT in PBS/BSA buffer. Finally, plasma membrane Nox4/8E9 mAb complex was revealed with 1h incubation at RT with a goat anti-mouse antibody conjugated to Alexa 633 diluted 1/200 in PBS/BSA buffer. For Nox4/p22^phox^ heterodimer formation study, C-20/A4 chondrocytes were permeabilized for 10 minutes with 0.1% (v/v) Triton X-100 and were stained for 1h with 5 µg of 16G6 mAb directed against p22^phox^ [30] in 200 µl PBS/BSA buffer. After two washes, p22^phox^ staining was revealed with a goat anti mouse antibody labeled Alexa 546 (red). In all experiments, cell nuclei were stained with Hoechst 33258 (0.5 µg/ml) and mounted in DABCO solution; they were sealed, and stored at 4°C in the dark. Confocal microscopy was carried out by using the Zeiss LSM510 NLO META. The pinhole was adjusted to 1 Airy unit resulting in 0.7 µm thick slices. Hoechst fluorescence was visualized using a 2P excitation.

### Statistical Data

Data are presented as means +/− S.D., significance levels are assessed using Student's paired t test. A p-value of 0.05 or less between groups is considered to indicate a statistically significant difference.

## Results

### Nox4 Mediates IL-1ß-effects on MMP-1 Secretion and DNA Fragmentation in C-20/A4 Chondrocyte Cells

**Figure 1:**
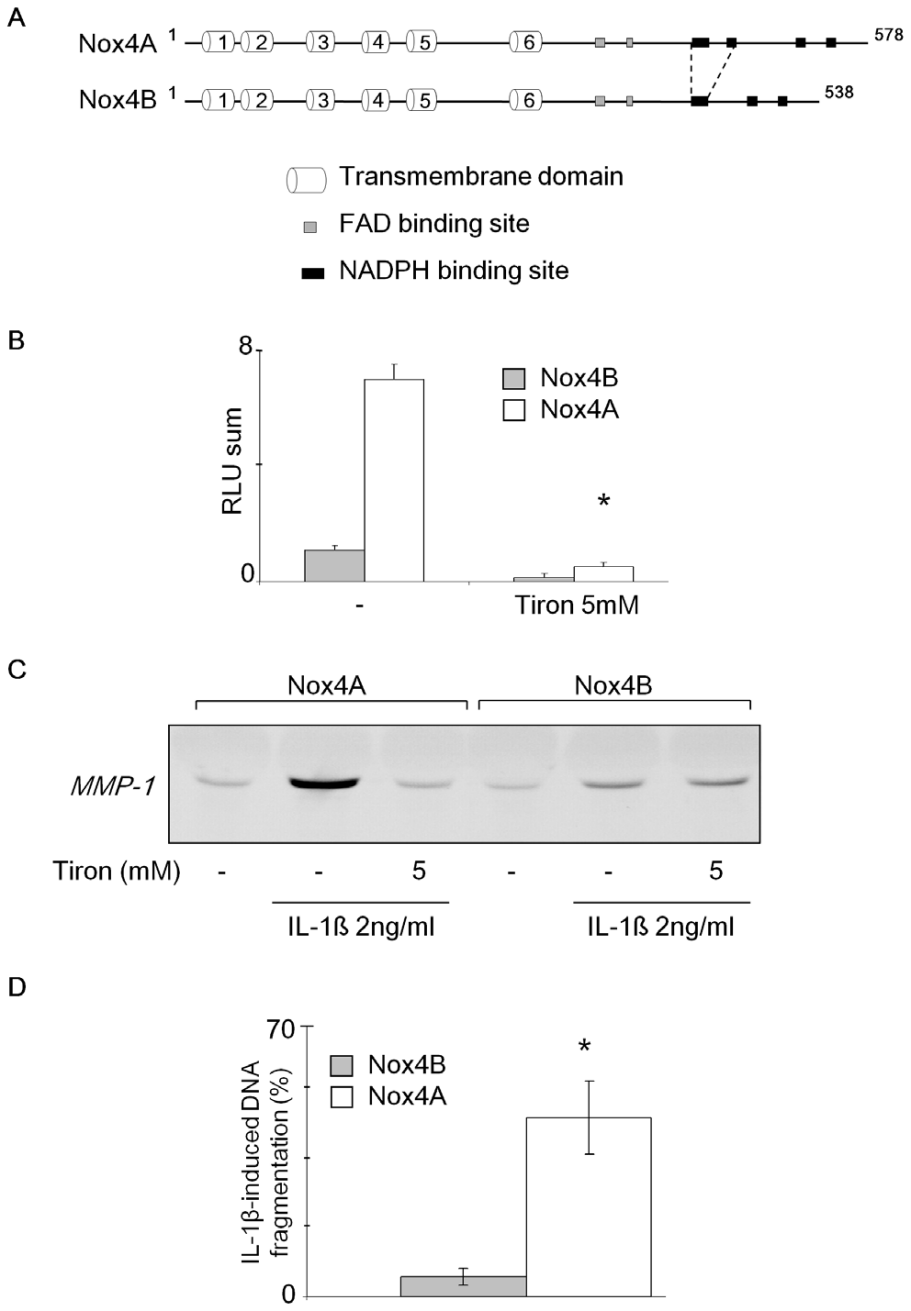
Nox4 mediates IL-1ß-effects on MMP-1 secretion and DNA fragmentation in C-20/A4 chondrocyte cells. (**A**) Predicted linear domain structure of Nox4A, the functional isoform and Nox4B, deleted for a putative NADPH binding site. (**B**) ROS production was measured by chemiluminescence on 5×10^5^ intact C-20/A4 chondrocytes overexpressing Nox4A or Nox4B treated or not with 5 mM of Tiron. Results are expressed as the sum of all RLU measurements recorded every 30 s during 45 min. Values represent the mean +/− S.D. of four determinations obtained the same day. * p<0.05 versus untreated Nox4A cells. (**C**) C-20/A4 chondrocytes transfected with genes encoding for Nox4A or Nox4B were stimulated or not with 2 ng/ml IL-1ß with or without 5 mM Tiron. After 48h, supernatant was collected, concentrated 10 times by centricon and 10 µg of proteins were loaded for MMP-1 immunodetection by Western Blot. Results are representative of three independent experiments. (**D**) C-20/A4 chondrocytes transfected with genes encoding for Nox4A or Nox4B were treated or not with 10 ng/ml IL-1ß. After 5 days, cells were detached and fixed with ice cold absolute ethanol. Cells were then washed twice in PBS and stained with propidium iodide before flow cytometry acquisition. * p<0.05 versus Nox4B cells.

Our previous studies showed that Nox4 is involved in MMP-1 expression in C-20/A4 human chondrocyte cells [22]. To assess the role of Nox4 in chondrocytes, we generated a C-20/A4 chondrocyte cell line that provided a stable expression of Nox4A (Genbank No.AF254621), or Nox4B (Genbank No. AY288918) (Figure 1A). Nox4A correspond to the commonly described wild type Nox4. Nox4B is a splice variant in which the first NADPH binding site is missing and corresponds to a non functional enzyme. It has been described as a dominant negative variant in A549 cells [31]. Results showed a significant production of ROS by luminescence in Nox4A transfected cells compared with the cells that overexpress Nox4B (Figure 1B). Moreover, the ROS produced was neutralized by Tiron, an antioxidant molecule. After 24 h stimulation by 2 ng/ml IL-1β, a 12 fold increase of MMP-1 expression was observed by Western Blot in the media supernatant of the Nox4A transfected C-20/A4 chondrocytes (Figure 1C). Interestingly, the addition of 5 mM Tiron completely inhibits the effect of IL-1β on MMP-1 expression, strengthening the role of ROS on MMP-1 induced expression. Contrary to Nox4A, Nox4B transfected chondrocytes slightly increased by 3 fold the secretion of MMP-1 upon IL-1β stimulation. These data confirmed our previous observation that MMP-1 synthesis in C-20/A4 chondrocytes upon IL-1β signalling is mediated by Nox4 oxidase activity [22]. As reported, IL-1β triggers chondrocyte apoptosis [32]. We next investigate the DNA fragmentation level and found a 80% increase of cell death (SubG1 population) of Nox4A transfected chondrocytes by flow cytometry after 5 days incubation with 10 ng/ml IL-1β compared to the control chondrocytes transfected with Nox4B gene (Figure 1D). The scavenging of ROS production by N-acetyl cystein prevented the observed cell death (Figure S1A). Consistently, the exposure of WT chondrocytes to either H_2_O_2_ (Figure S1B left panel) or IL-1β (Figure S1B right panel) led to an increase of DNA fragmentation in a dose dependent manner that is inhibited by the addition of catalase PEG. All together, these data confirm that ROS and more specifically H_2_O_2_ produced by Nox4A upon IL-1β stimulation induced C-20/A4 chondrocyte cell death and MMP-1 secretion.

### HO-1 Overexpression Decreases Nox4 Activity in C-20/A4 Chondrocytes

**Figure 2:**
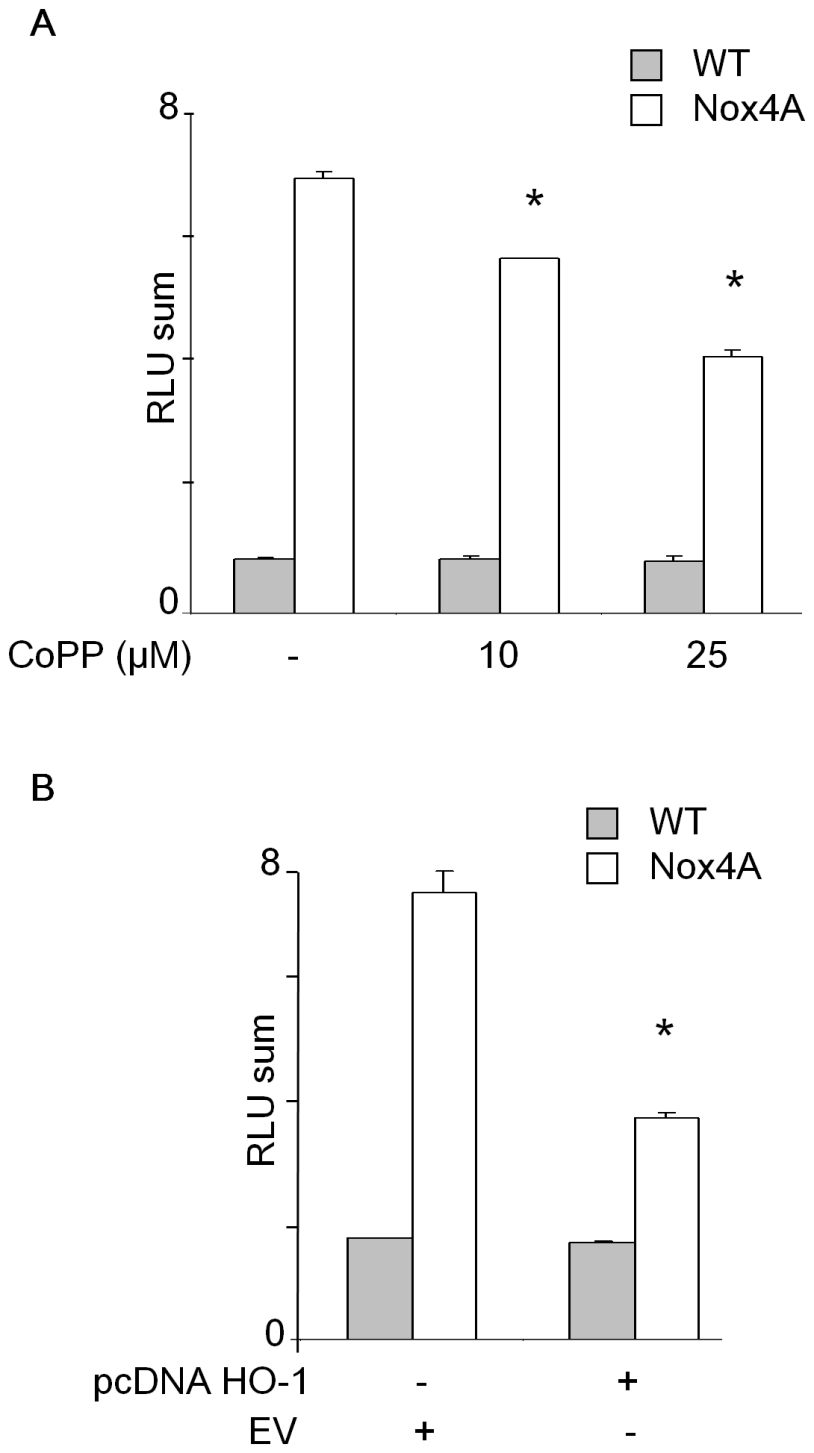
HO-1 overexpression decreases Nox4 activity in C-20/A4 chondrocytes. (**A**) C-20/A4 chondrocytes were treated or not with 10 or 25 µM CoPP-IX for 48 h. Nox4 activity was then measured by chemiluminescence from 5×10^5^ WT or Nox4A transfected cells. RLU were recorded every 30 s for 45 min as described in material and methods. * p<0.05 versus untreated Nox4 cells. (**B**) Similar experiments were also performed on chondrocytes (WT and Nox4A) stably transfected with HO-1 or the empty vector (EV) as described in material and methods. Values represent the mean +/− S.D. of three determinations obtained the same day and are representative of three independent experiments. * p<0.05 versus EV Nox4 cells.

Heme molecules (heme A and B) are essential to NADPH oxidase activity [33]. Therefore, decreasing of heme availability by modulating of HO-1 expression may affect Nox4 activity. We found that HO-1 expression was dose-dependently induced in C-20/A4 chondrocytes (Figure S2A) and HEK293 T-REx™ (Figure S2C) cells after 48 h incubation with 10 or 25 µM cobalt protoporphyrin-IX (CoPP-IX). Interestingly, incubation with CoPP-IX consequently decreased the ROS production in Nox4A chondrocytes (Figure 2A). Similar reduction (by 50%) of ROS production was obtained when HO-1 protein was overexpressed in Nox4A C-20/A4 chondrocytes (Figure 2B and S2B). To confirm that the observed effect of HO-1 was specifically related to Nox4 protein, we used another cellular model, HEK293 T-REx™ Nox4 cells, in which the expression of Nox4 is induced by the addition of tetracycline [28]. HO-1 expression was chemically induced by CoPP-IX in HEK293 T-REx™ Nox4 cells (Figure S2C) and the results confirmed a CoPP-IX dependent decrease of Nox4 activity up to 80% after 72 h HO-1 induction (Figure S3A). We confirmed those results by using the Amplex Red assay, another ROS detection method (Figure S3B). These data indicate that HO-1 act as a negative regulator of Nox4 activity in those two different cellular models.

### HO-1 Decreases MMP-1 Expression and Chondrocytes DNA Fragmentation

**Figure 3:**
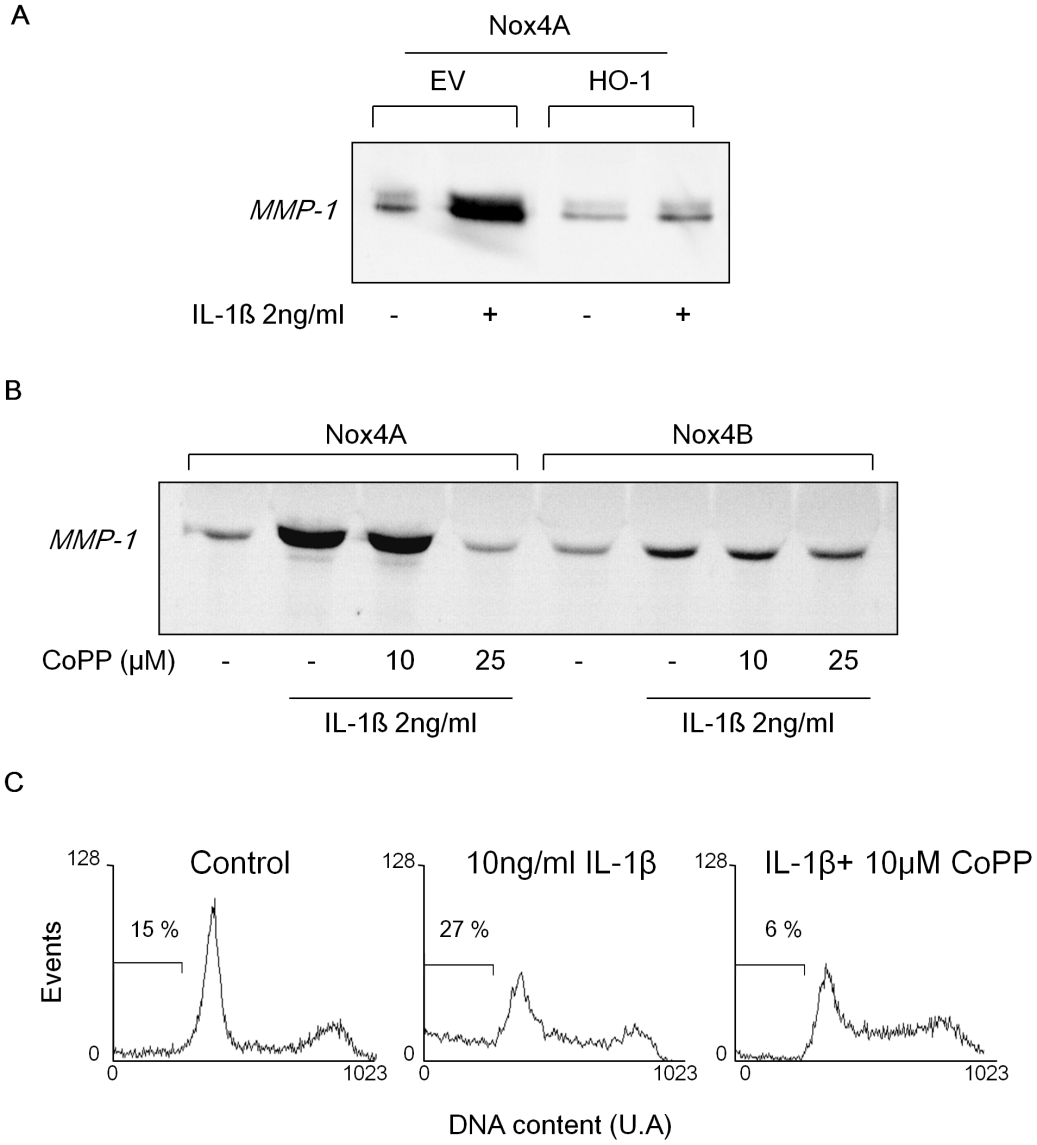
HO-1 decreases MMP-1 expression and chondrocytes DNA fragmentation. (**A** and **B**) C-20/A4 chondrocytes overexpressing Nox4A or Nox4B were induced or not for HO-1 expression with 10 or 25 µM CoPP-IX for 48h (A). C-20/A4 Nox4A chondrocytes were stably transfected with HMOX1 gene (encoding for HO-1) or the empty vector (EV) (B). Cells were stimulated or not with 2 ng/ml IL-1ß. After 48h, the media supernatant was collected, concentrated 10 times by centricon. 10 µg of proteins were loaded on 10% SDS-PAGE for MMP-1 immunodetection by Western Blot. (**C**) C-20/A4 Nox4A chondrocytes were treated or not with 10 ng/ml IL-1ß +/−10 µM CoPP-IX for 5 days in DMEM 2% fetal bovine serum. Cells were then washed, fixed with ice cold ethanol, stained with propidium iodide and 5×10^5^ cells fluorescence was assessed by FACS. Results are representative of three independent experiments.

We have shown that ROS generated by Nox4 are directly involved in the expression of MMP-1 and cell death in the C-20/A4 chondrocyte cells. We next investigated whether the increase of HO-1 expression could affect MMP-1 secretion induced by IL-1β. HO-1 expression was induced by 48 h incubation with CoPP-IX or by stable HO-1 gene transfection in Nox4A transfected chondrocytes. The amount of MMP-1 secreted in the media supernatant was evaluated by Western Blot after 48h stimulation by IL-1β. The overexpression of HO-1 by transfection reduced 4 times the quantity of MMP-1 in the media supernatant of Nox4A chondrocytes compared to control EV transfected cells (Figure 3A). Consistently, the HO-1 induction by CoPP-IX totally abolished the MMP-1 secretion and DNA fragmentation mediated by Nox4A upon IL-1β treatment. Interestingly, similar but milder significant effects were also observed on WT cells, probably due to the presence of the endogenous Nox4 protein (Figure S4). By contrast, no significant effects of HO-1 were noticeable on MMP-1 secreted by cells expressing Nox4B suggesting a potential dominant negative effect of this splice variant (Figure 3B). Together, these data confirm that the regulation of Nox4 activity by HO-1 influences directly MMP-1 secretion as well as chondrocytes death.

### Heme Availability does not Modify Nox4 Total Protein Expression but Affects Nox4 Subcellular Compartmentation

**Figure 4:**
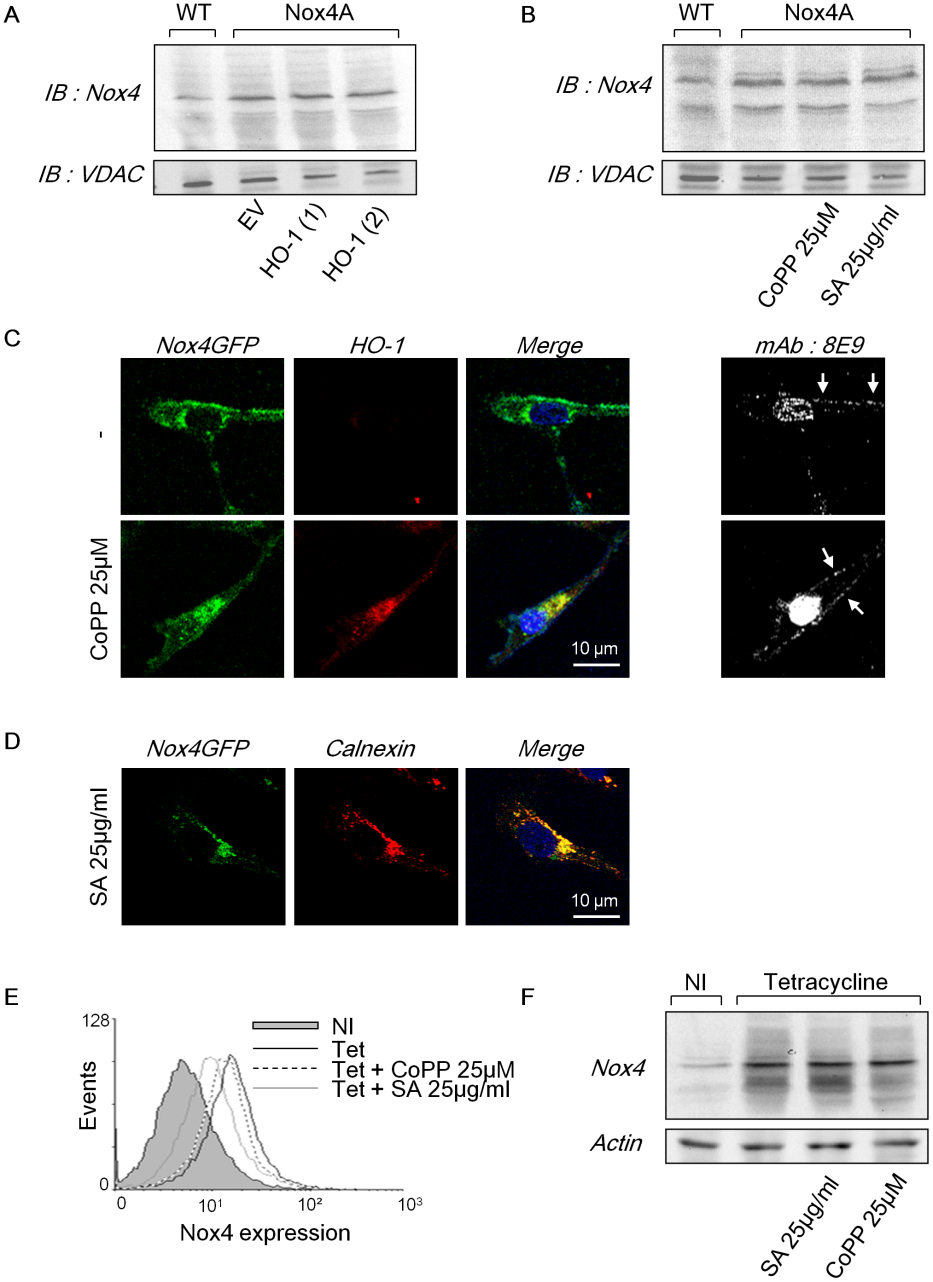
Heme availability does not modify Nox4 total protein expression but affects Nox4 subcellular compartmentation. (**A** and **B**) C-20/A4 Nox4A chondrocytes were transfected with HO-1 encoding vector or the empty vector (EV) ((1) and (2) are from two independent stable transfections) (**A**) or were treated with 25 µM CoPP-IX or 25 µg/ml SA during 48h (**B**). Nox4 expression was then assessed by Western Blot with 8E9 antibody on 150 µg of protein from 1% (v/v) Triton X-100 extract. VDAC protein served as loading control. (**C** and **D**) Nox4GFP overexpressing C-20/A4 chondrocytes were treated or not with 25 µM CoPP-IX (**C**) or 25 µg/ml succinylacetone (**D**) for 48h. (**C**) Cells were fixed with 4% paraformaldehyde (PFA) and plasma membrane Nox4 was stained with 8E9 mAb before cell permeabilization. Cells were then permeabilized with 0.1% (v/v) Triton X-100 and immunostained for HO-1 (red). Right panels show the 8E9 mAb immunostaining (white arrows) with the nucleus. (**D**) SA treated Nox4GFP expressing chondrocytes were fixed with 4% PFA and permeabilized with 0.1% (v/v) Triton X-100. Cells were then immunolabelled with calnexin (red) and nucleus was stained with Hoechst 33258 (blue). (**E** and **F**) HEK293 T-REx™ cells were induced or not for Nox4 expression with tetracycline (Tet) and for HO-1 with 25 µM CoPP-IX or were treated with 25 µg/ml SA during 48 h. (**E**) Nox4 expressed at the plasma membrane was stained with 8E9 mAb (recognizing an extracellular epitope of Nox4). Plasma membrane Nox4 expression was then quantified by flow cytometry. Non-induced (NI) cells are displayed in grey solid line, tet-induced cells in black continuous line, tet-induced cells treated by CoPP-IX in black dotted line and tet-induced cells treated with SA in grey continuous line. (**F**) Total Nox4 expression was assessed by Western Blot with 8E9 antibody on 150 µg of protein from a 1% (v/v) Triton X-100 extract. Actin protein served as loading control. Results are representative of three independent experiments.

Heme molecules are necessary for Nox2 dimerization with its stabilizing subunit p22^phox^ and maturation, and for plasma membrane addressing of the mature heterodimer (cytochrome *b*_558_) in PLB-985 cells [25]. In the absence of hemes, Nox2 is unstable and is degraded by the proteasome [26]. Thus, we evaluated the molecular impact of heme catabolism by HO-1 on Nox4 expression. We did not observed significant changes in Nox4 expression when HO-1 was overexpressed either by transfection or by CoPP-IX induction in both C-20/A4 and HEK293 T-REx™ Nox4 cell types compared to untreated cells (Figure 4A, 4B and 4F). These results therefore demonstrate that HO-1 decreases Nox4 activity without affecting significantly its expression. Given the extremely short half-life of superoxide that dismutes quickly into H_2_O_2_, the site of its production determines its biological impact. The effect of HO-1 on Nox4 subcellular localization was assessed in the C-20/A4 chondrocytes. Consistent with data shown in HEK293 cells [27], transfection of Nox4GFP fusion gene led to endoplasmic reticulum (ER) and plasma membrane localization of the fusion protein in C-20/A4 chondrocytes by confocal microscopy (Figure 4C). Plasma membrane localization of Nox4GFP was confirmed by using 8E9 antibody that recognizes an extracellular epitope of Nox4 protein [27] and that was added before cell permeabilization (Figure 4C, right panel). After 48h incubation with 25 µM CoPP-IX, a clear induced expression of HO-1 was evidenced in C-20/A4 chondrocytes that was co-localized with Nox4GFP fusion protein (Figure 4C). We observed that upon 25 µM CoPP-IX incubation, Nox4 remained expressed at the plasma membrane as illustrated by the 8E9 mAb staining (Figure 4C, right panel). Interestingly, after treatment with the heme synthesis inhibitor succinylacetone (SA), the total amount of Nox4 protein was not affected (Figure 4B) whereas its plasma membrane localization seemed compromised since a large majority of Nox4GFP proteins remained in the ER and were co-localized with calnexin, an endoplasmic reticulum marker (Figure 4D).

To confirm those observations, we used another cellular model HEK 293 T-REx™ (Figure 4E and 4F). Nox4 subcellular localization was assessed by flow cytometry using the 8E9 mAb on unpermeabilized tetracycline induced T-REx™ Nox4A cells. We observed that Nox4 expression at the plasma membrane after 48h tetracycline induction was not affected by the increase of HO-1 expression upon 48h CoPP-IX treatment (Figure 4E). On the contrary, 24h treatment with SA decreased by 60% the plasma membrane expression of Nox4 (Figure 4E) without affecting the total amount of Nox4 protein (Figure 4F). These data together suggest that the inhibition of heme synthesis by SA treatment rather than the catabolism of heme mediated by HO-1 affects plasma membrane localization of Nox4GFP which remains confined in the synthesis compartment (ER).

### Inhibition of Heme Synthesis, Rather than Heme Catabolism by HO-1, Alters Nox4/p22^phox^ Dimer Formation and Heme Integration

**Figure 5:**
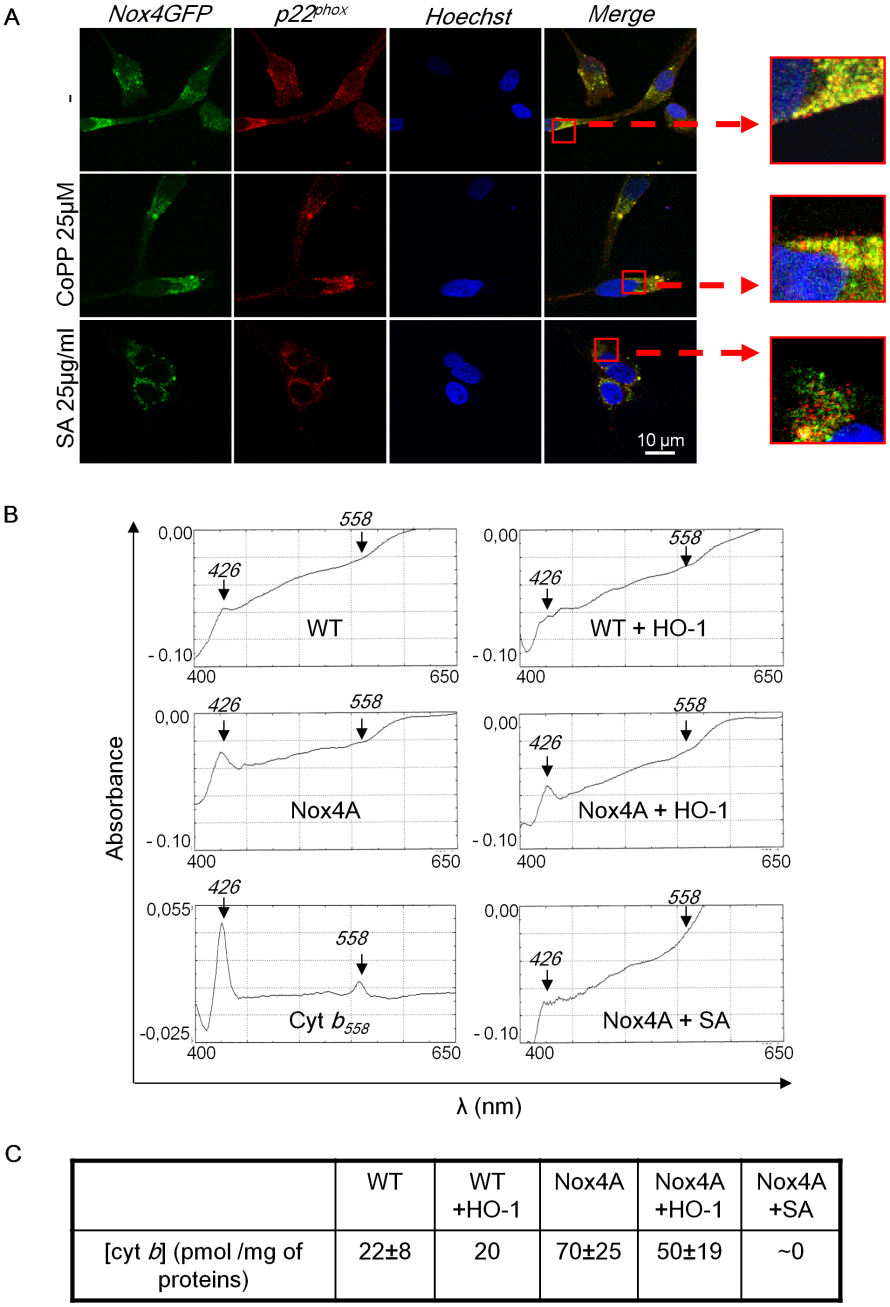
Inhibition of heme synthesis, rather than heme catabolism, affects Nox4/p22 dimer formation and heme integration. (**A**) Nox4GFP C-20/A4 chondrocytes were treated with 25 µM CoPP-IX or 25 µg/ml SA during 48 h. Cells were then fixed with PFA, permeabilized and p22^phox^ was stained with 16G7 mAb antibody (red). The nucleus was stained with Hoechst 33256 (blue). (**B**) WT and Nox4 chondrocytes were treated or not with succinylacetone (25 µg/ml during 48 h) or were transfected with HO-1. Reduced minus oxidized difference spectra of Nox4 were assessed on 5 mg/ml of proteins from a 1% (v/v) Triton X-100 extracts. The positive control was obtained with cytochrome *b*_558_ purified from human neutrophils. (**C**) Concentration of cytochrome *b* in the extract was calculated with a ε_426_ nm value of 106 mM^−1^ cm^−1^.

Since cellular heme content influences Nox4 compartmentation and potentially its maturation, the consequence of a modulation of heme amount on Nox4/p22^phox^ heterodimer formation was investigated (Figure 5). In absence of HO-1, confocal images showed a strict co-localization of Nox4GFP fusion protein with p22^phox^ in C-20/A4 chondrocytes arguing in favour of the presence of a mature Nox4/p22^phox^ complex (Figure 5A). Interestingly, SA treatment abolished this co-localization whereas the induction of HO-1 by CoPP-IX did not (Figure 5A). We next assessed the spectral properties of Nox4/p22^phox^. The Nox2/p22^phox^ heterodimer, named cytochrome *b*_558_ is characterized by a redox spectrum with three absorption bands at 426, 530 and 558 nm. Due to a high homology between Nox4 and Nox2, the Nox4/p22^phox^ heterodimer may display similar spectral properties [22]. 1% (v/v) Triton X-100 protein extracts from WT, Nox4A or Nox4A HO-1 transfected C-20/A4 chondrocytes were prepared. Reduced minus oxidized difference spectra were measured (Figure 5B). Similar to cyt *b_558_*, results showed a slight peak at 426 nm (the Soret Band) in WT chondrocytes that was significantly enhanced (3.5 fold) in chondrocytes overexpressing Nox4A. To verify that this increase in absorbance at 426 nm was specific to Nox4 overexpression and not from a mitochondria contamination, we evaluated the expression level of VDAC, a mitochondria marker. We found that VDAC level was even in all samples, resulting in an increase of Abs_426nm_/VDAC ratio in protein extract from Nox4A chondrocytes compared with protein extracts from WT (Figure S5). In the absence of a known molar extinction coefficient at 426 nm (ε_426_) value for Nox4, we estimated the amount of Nox4/p22^phox^ dimer (cyt *b*) of Nox4 using the ε_426_ value of the cytochrome *b_558._* 22 pmol of cyt *b* per mg protein extract was initially found in WT cells and was increased up to ∼70 pmol cyt *b* per mg of proteins in Nox4 transfected cells (Figure 5B and 5C). In addition, overexpression of HO-1 slightly decreased the cyt *b* amount (from 70 to 50 pmol/mg of protein). Conversely, no spectra were observed upon SA treatment (Figure 5B and 5C) which is correlated to an absence of ROS production under those conditions (unpublished data). Taken together, these data show that the presence of heme molecules is necessary for the Nox4/p22^phox^ heterodimerization. However, despite a significant inhibitory effect of HO-1 on Nox4 activity, the maturation of the Nox4/p22^phox^ heterodimer appears not significantly affected by HO-1.

### Carbon Monoxide Decreases Nox4 Activity and MMP-1 Expression

**Figure 6:**
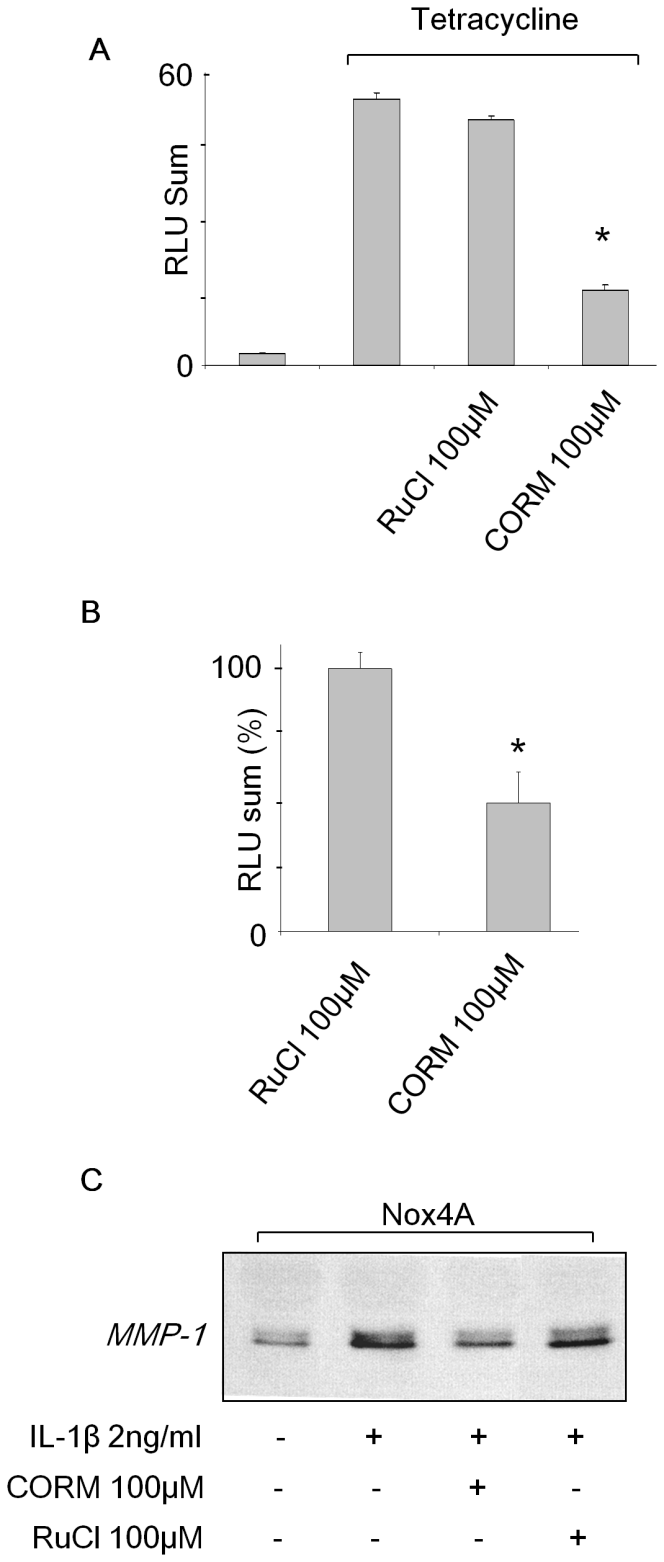
Carbon monoxide (CO) decreases Nox4 activity and MMP-1 expression. (**A**) Tet induced HEK293 T-REx™ Nox4 cells were treated for 1h with 100 µM CORM (a CO donor) or RuCl (the control molecule). Histogram shows the RLU sum obtained from the 45 min kinetic of Nox4 activity from 5×10^5^ cells assessed by chemiluminescence. * p<0.05 versus RuCl treated cells. (**B**) 5×10^5^ C-20/A4 chondrocytes were pre-treated for 1 h with 100 µM CORM or RuCl. Nox4 activity was then assessed by chemiluminescence. Results are expressed as percentage of RuCl treated cells. Values represent the mean +/− S.D. of determinations in triplicate obtained the same day. * p<0.05 versus RuCl treated cells. (**C**) C-20/A4 Nox4A chondrocytes were stimulated with 2 ng/ml IL-1β and treated with 100 µM CORM or the control RuCl for 16 h. Medium supernatant was then collected and concentrated 10 times by centricon. 10 µg of proteins were loaded on 10% SDS-PAGE for MMP-1 immunodetection by Western Blot. Results are representative of three independent experiments.

A possible mechanism of action of HO-1 on Nox4 activity could be mediated by heme degradation by-products (Carbon monoxide (CO), Biliverdin (BV) and Bilirubin (BR)). The consequences of CO have been studied on Nox1 and Nox2 activity [34], [35], [36] but not on that of Nox4 so far. In order to investigate whether CO interfere with Nox4 activity, we evaluated the effects of CORM-II, a CO donor [37], on tetracycline induced HEK293 T-REx™ Nox4 cells. CORM-II decreased significantly (by 75%) the production of ROS whereas the control RuCl compound which do not release CO had no effect (Figure 6A). To avoid a potential antioxidant property mediated by CORM-II itself independently of the presence of CO, CORM-II was prepared extemporaneously or 24 h before (time necessary to CO to totally volatized) its supplementation to the cells. Indeed, a significant decrease of Nox4 activity was only observed with 50 µM CORM prepared extemporaneously and not with those prepared 24 h earlier (Figure S6). The inhibitory effect of CO was confirmed on Nox4A transfected C-20/A4 chondrocytes in which CORM-II decreased significantly the ROS production by 50% (Figure 6B).

Finally, we tested whether the inhibition of Nox4 activity by CO could prevent the MMP-1 secretion induced by IL-1β. For this we treated IL-1β-induced Nox4A transfected C-20/A4 cells with 100 µM of CORM-II or RuCl (control) and evaluated the amount of MMP-1 secreted. CORM-II reduced by approximately half the quantity of MMP-1 secreted in the media supernatant compared to the control RuCl (Figure 6C) strengthening the link in the IL-1β induced pathway between HO-1, CO, Nox4 activity, MMP-1 secretion and DNA fragmentation.

## Discussion

Physiological relevance of ROS in chondrocyte physiology remains elusive. ROS might play an important role in chondrogenesis [13], [14]. Indeed, previous works have reported a role for ROS in mediating chondrocyte hypertrophy during endochondral ossification. However, hypertrophic phenotype is also observed among osteoarthritic chondrocytes which die by apoptosis [12] and NADPH oxidase Nox4 may contribute to this phenomenon [13]. Furthermore, pseudoachondroplasia, a genetic pathology caused by a mutation on the protein COMP (Cartilage Oligo Matrix Protein) is characterised by abnormal joint architecture, joint erosion and osteoarthritis. Interestingly, a recent study showed a 100 fold increase of Nox4 mRNA in Rat Chondrosarcoma Cells (RCS) expressing mutated COMP [21]. Necroptosis, a caspase independent cell death process was triggered in chondrocytes following DNA damages induced by ROS. Therefore, while the implication of ROS in chondrocyte pathologies has been suggested, no studies on the specific role of NADPH oxidase Nox4 was proposed until yet.

The extracellular matrix catabolism and chondrocyte death are the two main causes leading to cartilage degradation and osteoarthritis. In this study, we demonstrated for the first time the involvement of Nox4 derived ROS in DNA fragmentation and chondrocyte death under IL-1β cytokinic stress. Moreover, we identify a mechanism of regulation of Nox4 activity by HO-1 through CO generation as by-product of heme degradation validated in two different cellular models. Our findings indicate that the use of the CO generation compound (CORM-II) or the induced expression of HO-1 down-regulates the ROS production by Nox4 in human C-20/A4 chondrocytes that consequently reduce MMP-1 secretion and cell death, two main features in osteoarthritis. Those observations raise the prospect that targeting Nox4 activity and HO-1 may represent novel therapeutic strategies in OA.

It is well known that Nox2 expression, heterodimerization with p22^phox^ and addressing to the plasma membrane are dependent on the presence of heme molecules [25]. In this work we studied the impact of heme metabolism on Nox4 expression, subcellular compartmentation and maturation by comparing the effect of HO-1 that catalyzes the degradation of heme to that of succinylacetone, a heme synthesis inhibitor. The localization at the plasma membrane is a good indication of the mature state for Nox2. Consistently with our previous observation in HEK293 T-REx™ cells [27], we found that functional Nox4 is partly expressed at the plasma membrane in C-20/A4 chondrocytes. Our data, showing that the inhibition of heme synthesis rather than heme degradation induces its retention in the endoplasmic reticulum and absence to the plasma membrane, indicate that heme incorporation by Nox4 is a prerequisite to its dimerisation and maturation. Indeed, a SA treatment eradicates totally the Nox4 redox differential spectrum measured indicating the absence of prosthetic heme group. A role of heme in Nox4/p22^phox^ heterodimerization was suggested by Ambasta and coworkers by substituting the histidine 115 with a Leucine [38]. This mutation located on a putative heme coordination site disrupted the Nox4/p22^phox^ heterodimer. Conversely, the induced expression of HO-1 is unable to remove significantly hemes incorporated in Nox4/p22^phox^ complex characterized by an intact differential spectrum. Here, we demonstrate that in absence of heme, Nox4/p22^phox^ maturation, plasma membrane addressing and NADPH oxidase activity are abolished without affecting the total expression of Nox4. This contrasts with the observation made by Taillé and co-workers showing that HO-1 decreases the heme bioavailability and consequently Nox2/p22^phox^ expression in RAW264.7 macrophages [39]. Our data are consistent with previous findings in rat aortic tissue [40], where the production of ROS was decreased without any change in both Nox2 and Nox4 expression after HO-1 induction by hemin. However, this *in vivo* study could not discriminate whether the source of ROS production is Nox2 or Nox4.

In this work we demonstrate that HO-1 is able to modulate Nox4 activity and validate that effect in two different cellular models. Indeed, upon induction of HO-1 expression with CoPP-IX or plasmid overexpression, a significant decrease of Nox4 oxidase activity was observed in the HEK293 T-REx™ cells and C-20/A4 chondrocytes. Similar data were previously reported in which the Heme oxygenase isoform HO-2 was shown to decrease Nox4 activity in microvasculature [41]. To our knowledge, it is the first time that HO-1 is reported as a regulator of Nox4 activity. We studied the HO-1 capability to inhibit Nox4 activity in the IL-1β pathway leading to events that participate to the progression of OA. We showed that inducing or overexpressing HO-1 proteins strongly reduces the matrix metalloproteinase MMP-1 secretion and chondrocyte cell death, therefore acting potentially to prevent cartilage degradation.

Unlike the SA treatment that abolishes Nox4 activity by affecting its heterodimerization and maturation, it is intriguing to observe that the induced expression of HO-1 inhibits Nox4 activity without affecting Nox4 synthesis pathway. This raises the hypothesis that HO-1 could interfere directly or indirectly with Nox4 activity itself. The cellular concentration of heme is depending on the equilibrium between expression of hemoproteins, heme synthesis and heme degradation catalyzed by Heme oxygenase-1 to gives rise to carbon monoxide, biliverdin and bilirubin (CO, BV and BR) [24]. Among them, CO was shown to modulate Nox1 and Nox2 activity [34], [35], [36]. Moreover, CO may decrease both ROS and NO production in osteoarthritic chondrocytes stimulated by IL-1β [37]. This was associated to a weak expression of MMP-1, MMP-3, MMP-10 and MMP-13 [42]. In our study, we showed that CO reduces Nox4 activity. At least other 3 studies suggested a direct binding of CO on Nox1 and Nox2 [34], [35], [36]. However, it was demonstrated the absence of binding of CO on cytochrome *b*_558_ [43]. Our data actually could not discriminate whether the impact of CO on Nox4 is direct or not; nevertheless, the CO donor CORM-II decreased significantly MMP-1 secretion and ROS production by IL-1β stimulated C-20/A4 chondrocytes.

Taken together, our results suggest that Nox4 is directly involved in MMP-1 expression and C-20/A4 chondrocyte death under IL-1β cytokinic stress. HO-1 is a negative modulator of Nox4 oxidase activity but does not significantly impact on its expression, subcellular localization and dimerization with its partner p22^phox^. On the other hand, inhibition of heme synthesis by succinylacetone affects profoundly maturation and function of Nox4. Therefore, inhibition of Nox4 activity by HO-1 is not due to the consequence of a decrease of heme bioavailability for Nox4, but mainly to CO production and its effect on oxidase activity.

These *in vitro* results highlight a possible role for Nox4 in OA, mediating pro-catabolic effect of IL-1β. Whether Nox4 is a physiological target or not in OA is currently under investigation in human primary chondrocytes. Nevertheless, homeostasis of the cartilage may depend on the balance between the expression of Nox4 and HO-1 and targeting those protein’s functions may provide therapeutic benefits for the treatment of articular conditions.

## Supporting Information

Figure S1 **Nox4 derived ROS mediate IL-1β induced cell death. (A)** Pictures taken under inverted microscope of C-20/A4 chondrocytes transfected with Nox4A or Nox4B encoding genes. Cells were treated with 10 ng/ml IL-1β, +/−500 nM N-acetyl cystein (NAC) during 5 days. Representative picture illustrating cell death by floating cell and low confluency compared to the untreated cells. (**B)** C-20/A4 WT chondrocytes were treated with increasing concentration of H_2_O_2_ (0; 100; 250 or 500 µM) or with IL-1ß (0; 2 or 10 ng/ml) +/− catalase-PEG 200 U. After 5 days, cells were detached and fixed with ice cold absolute ethanol. Cells were then washed twice in PBS and stained with propidium iodide before FACS acquisition.
(TIF)

Figure S2 **HO-1 is induced by CoPP-IX or plasmid transfection.** (**A**) C-20/A4 chondrocytes were treated 48 h with 10 or 25 µM CoPP-IX or (**B**) were stably transfected with HO-1 encoding plasmid. (**C**) HEK 293 T-REx™ Nox4 cells were treated 48 h with 10 or 25 µM CoPP-IX. (**A**, **B** and **C**) Cells were then lysed by using a Triton X-100 extract. HO-1 expression was assessed by Western Blot. (**D**) Confocal microscopy shows HO-1 immunostaining (red) of C-20/A4 chondrocytes induced or not for HO-1 expression with 25 µM CoPP-IX during 48 h. Results are representative of three independent experiments.
(TIF)

Figure S3 **HO-1 decreases Nox4 activity in HEK293 T-REx™ Nox4 cells.** (**A**) Cells were treated or not with CoPP-IX in order to induce HO-1 expression. Incubation time (48 or 72 h) and dose effect (10 or 25 µM) of CoPP-IX were assessed on ROS production by 5×10^5^ intact tet-induced cells. Results expressed the sum of all RLU measurements, acquired every 30s during 45 min. (**B**) Tet-induced HEK293 T-REx™ cells were incubated for 48 h with 10 or 25 µM CoPP-IX and total H_2_O_2_ production was assessed by the Amplex Red method on 5×10^5^ cells. Results expressed the sum of all RFU measurements, acquired every 2 min for 30 min. Values represent the mean +/− S.D. of four determinations obtained the same day and are representative of three independent experiments.
(TIF)

Figure S4 **Effects of HO-1 overexpression on MMP-1 secretion and cell death in WT C-20/A4. (A)** WT chondrocytes were induced for HO-1 expression with 10 or 25 µM CoPP-IX for 48h and stimulated or not with 2 ng/ml IL-1ß. After 48h, the media supernatant was collected, concentrated 10 times by centricon. 10 µg of proteins were loaded on 10% SDS-PAGE for MMP-1 immunodetection by Western Blot. (**B** and **C**) C-20/A4 WT cells were treated or not with 10 ng/ml IL-1ß +/−10 µM CoPP-IX for 5 days in DMEM 2% fetal bovine serum. (**B**) Cells were then washed, fixed with ice cold ethanol, stained with propidium iodide and 5×10^5^ cells fluorescence was assessed by FACS. (**C**) Culture supernatant was collected to assess cellular membrane integrity by measuring the cytosolic LDH activity. Results are representative of three independent experiments. * p<0.05 versus IL-1β treated cells.
(TIF)

Figure S5 **HO-1 does not change Nox4 expression and heme integration.** The graph shows the ratio between the absorbance at 426nm of the differential redox spectra and densitometric value of VDAC expression obtained by Western Blot on the same soluble extracts used in the Figure 5B. Results were generated from three independent experiments.
(TIF)

Figure S6 **CORM does not exhibit an antioxidant property.** The effect of 50 µM CORM extemporaneously prepared (t0h) versus prepared 24h before the experiment (t24h) in which all the CO is volatilized, was investigated on Tet-induced HEK293 T-REx™ cells. Nox4 activity was measured by chemiluminescence. * p<0.05 versus CORM t24h treated cells. Results are representative of three independent experiments.
(TIF)

Text S1 (DOC)
