# Supplementary figures and images for: Heme Oxygenase-1 Regulates Matrix Metalloproteinase MMP-1 Secretion and Chondrocyte Cell Death via Nox4 NADPH Oxidase Activity in Chondrocytes
Source: PLoS One. 2013 Jun 20;8(6):e66478. doi: 10.1371/journal.pone.0066478 (PMC3688771; doi:10.1371/journal.pone.0066478)

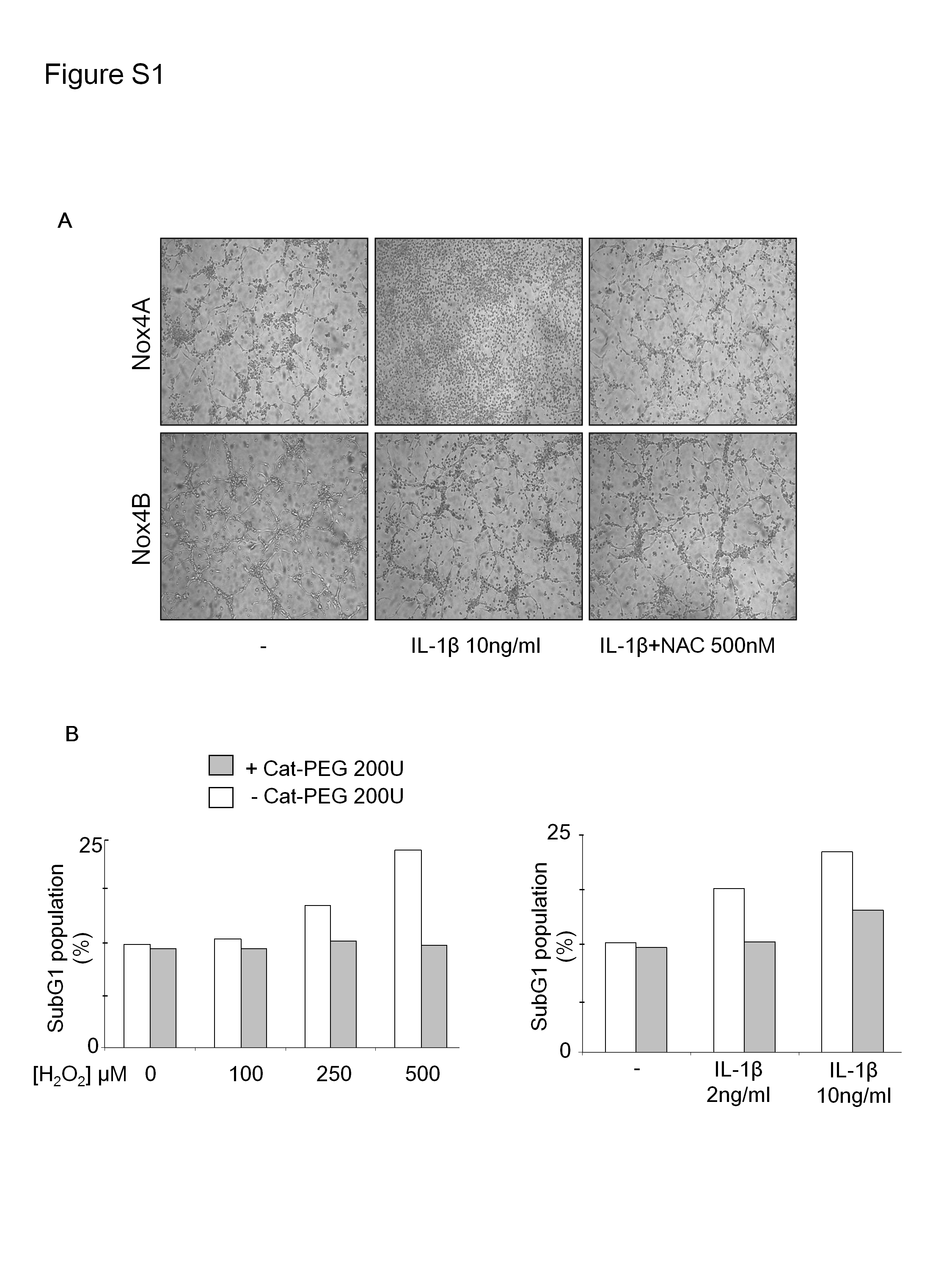

Supplement: Figure S1 — Nox4 derived ROS mediate IL-1β induced cell death. (A) Pictures taken under inverted microscope of C-20/A4 chondrocytes transfected with Nox4A or Nox4B encoding genes. Cells were treated with 10 ng/ml IL-1β, +/−500 nM N-acetyl cystein (NAC) during 5 days. Representative picture illustrating cell death by floating cell and low confluency compared to the untreated cells. (B) C-20/A4 WT chondrocytes were treated with increasing concentration of H2O2 (0; 100; 250 or 500 µM) or with IL-1ß (0; 2 or 10 ng/ml) +/− catalase-PEG 200 U. After 5 days, cells were detached and fixed with ice cold absolute ethanol. Cells were then washed twice in PBS and stained with propidium iodide before FACS acquisition. (TIF) [file pone.0066478.s001.tif]

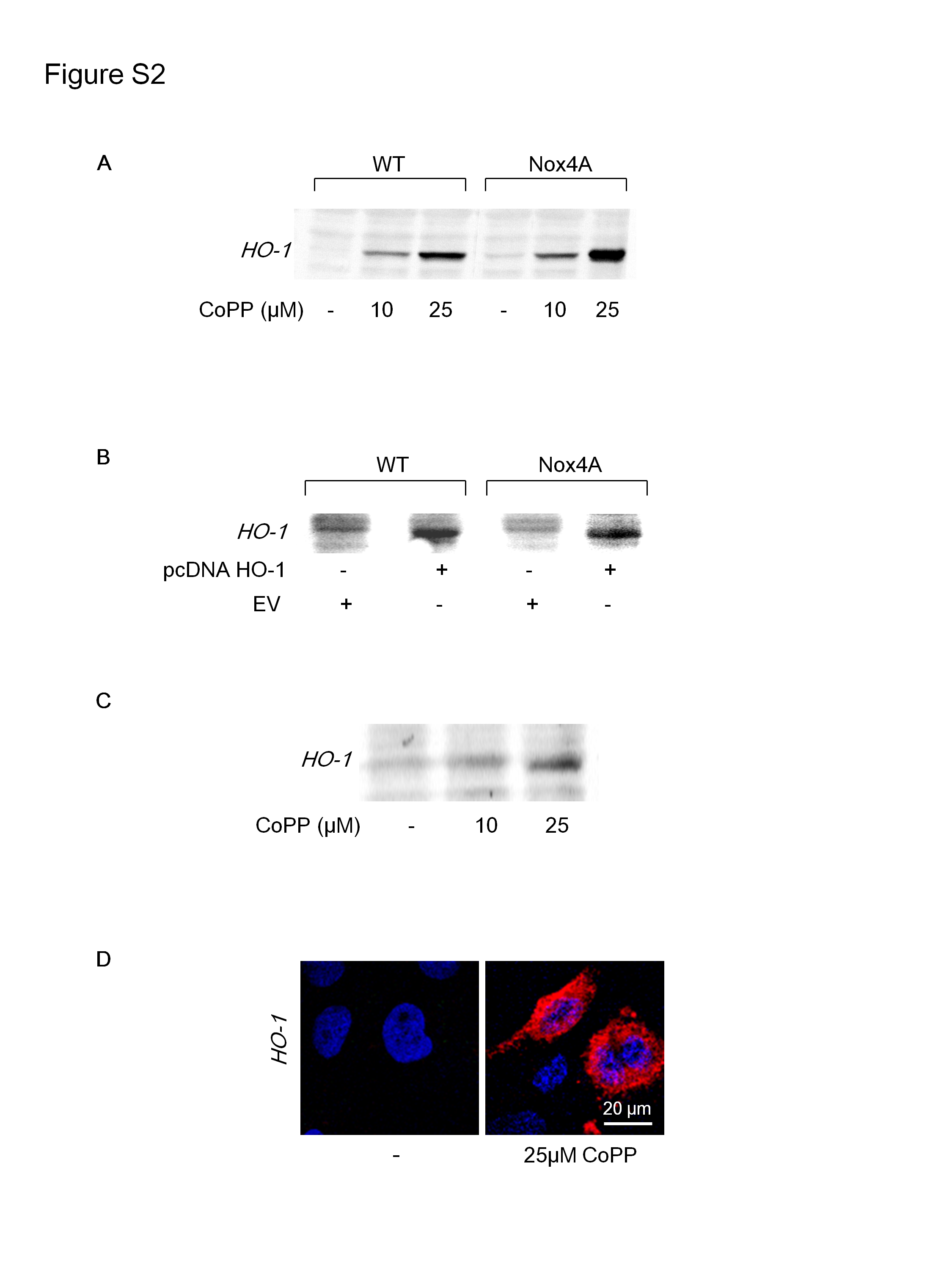

Supplement: Figure S2 — HO-1 is induced by CoPP-IX or plasmid transfection. (A) C-20/A4 chondrocytes were treated 48 h with 10 or 25 µM CoPP-IX or (B) were stably transfected with HO-1 encoding plasmid. (C) HEK 293 T-REx™ Nox4 cells were treated 48 h with 10 or 25 µM CoPP-IX. (A, B and C) Cells were then lysed by using a Triton X-100 extract. HO-1 expression was assessed by Western Blot. (D) Confocal microscopy shows HO-1 immunostaining (red) of C-20/A4 chondrocytes induced or not for HO-1 expression with 25 µM CoPP-IX during 48 h. Results are representative of three independent experiments. (TIF) [file pone.0066478.s002.tif]

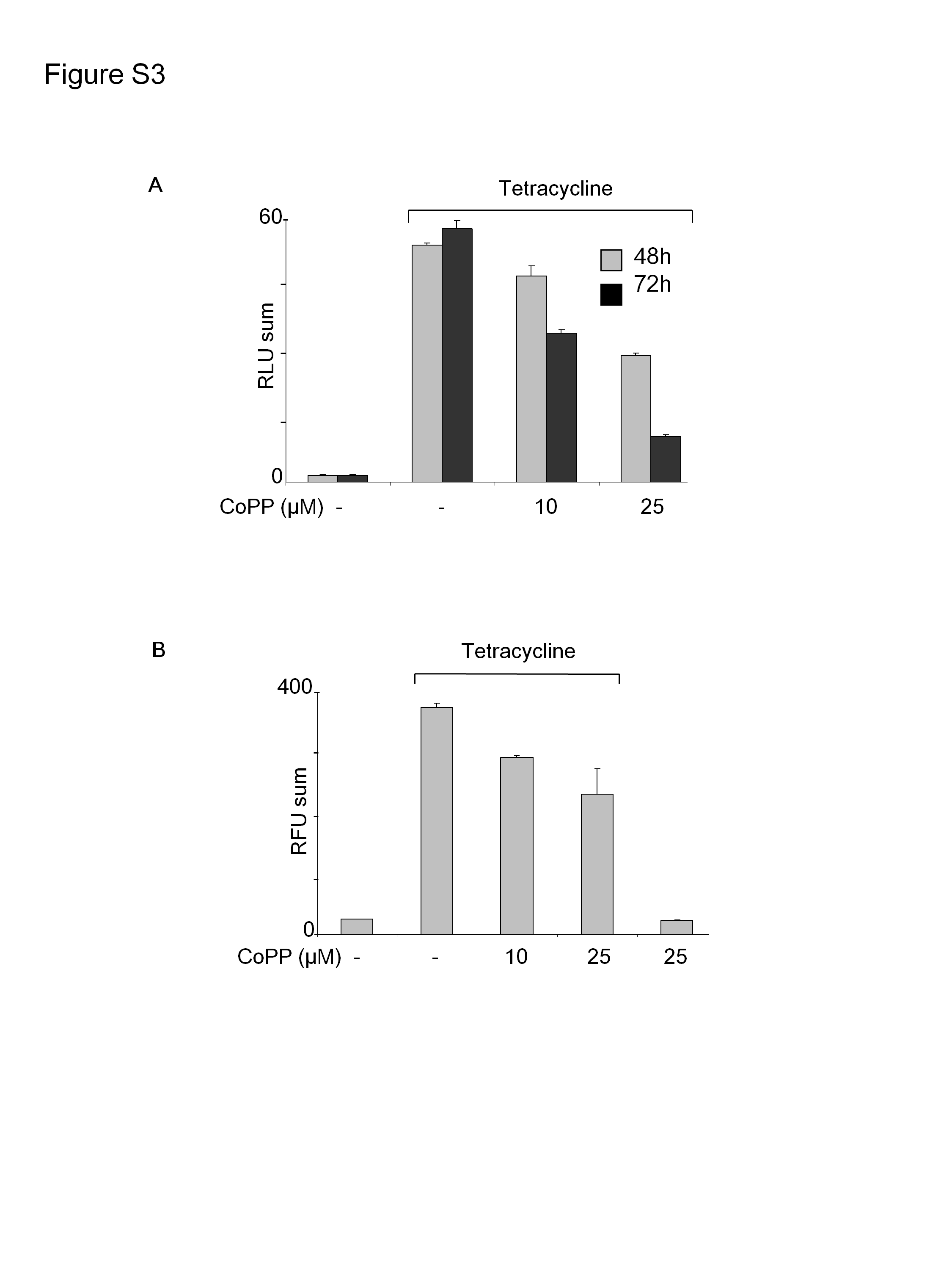

Supplement: Figure S3 — HO-1 decreases Nox4 activity in HEK293 T-REx™ Nox4 cells. (A) Cells were treated or not with CoPP-IX in order to induce HO-1 expression. Incubation time (48 or 72 h) and dose effect (10 or 25 µM) of CoPP-IX were assessed on ROS production by 5×10^5^ intact tet-induced cells. Results expressed the sum of all RLU measurements, acquired every 30s during 45 min. (B) Tet-induced HEK293 T-REx™ cells were incubated for 48 h with 10 or 25 µM CoPP-IX and total H2O2 production was assessed by the Amplex Red method on 5×10^5^ cells. Results expressed the sum of all RFU measurements, acquired every 2 min for 30 min. Values represent the mean +/− S.D. of four determinations obtained the same day and are representative of three independent experiments. (TIF) [file pone.0066478.s003.tif]

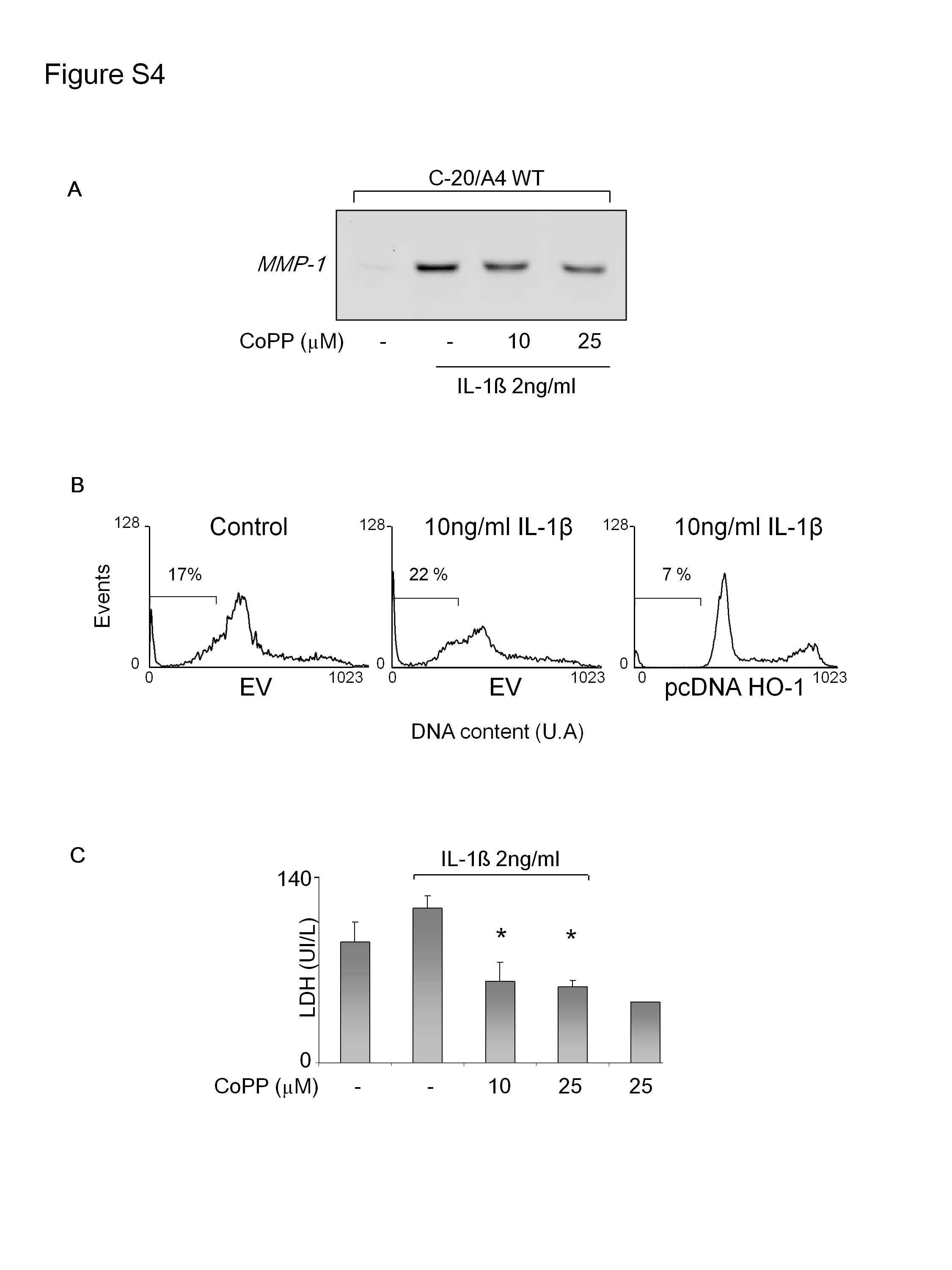

Supplement: Figure S4 — Effects of HO-1 overexpression on MMP-1 secretion and cell death in WT C-20/A4. (A) WT chondrocytes were induced for HO-1 expression with 10 or 25 µM CoPP-IX for 48h and stimulated or not with 2 ng/ml IL-1ß. After 48h, the media supernatant was collected, concentrated 10 times by centricon. 10 µg of proteins were loaded on 10% SDS-PAGE for MMP-1 immunodetection by Western Blot. (B and C) C-20/A4 WT cells were treated or not with 10 ng/ml IL-1ß +/−10 µM CoPP-IX for 5 days in DMEM 2% fetal bovine serum. (B) Cells were then washed, fixed with ice cold ethanol, stained with propidium iodide and 5×10^5^ cells fluorescence was assessed by FACS. (C) Culture supernatant was collected to assess cellular membrane integrity by measuring the cytosolic LDH activity. Results are representative of three independent experiments. * p<0.05 versus IL-1β treated cells. (TIF) [file pone.0066478.s004.tif]

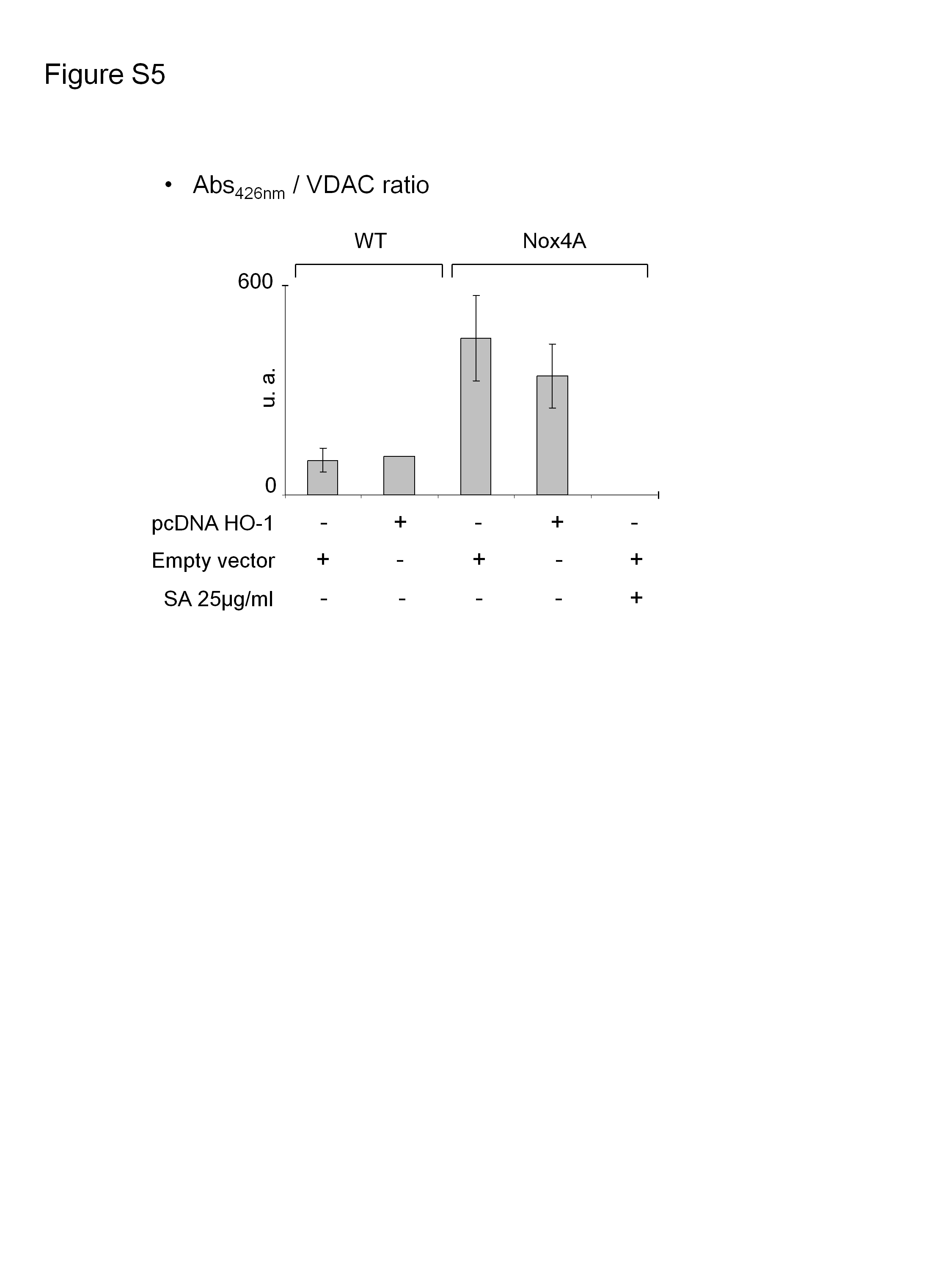

Supplement: Figure S5 — HO-1 does not change Nox4 expression and heme integration. The graph shows the ratio between the absorbance at 426nm of the differential redox spectra and densitometric value of VDAC expression obtained by Western Blot on the same soluble extracts used in the Figure 5B. Results were generated from three independent experiments. (TIF) [file pone.0066478.s005.tif]

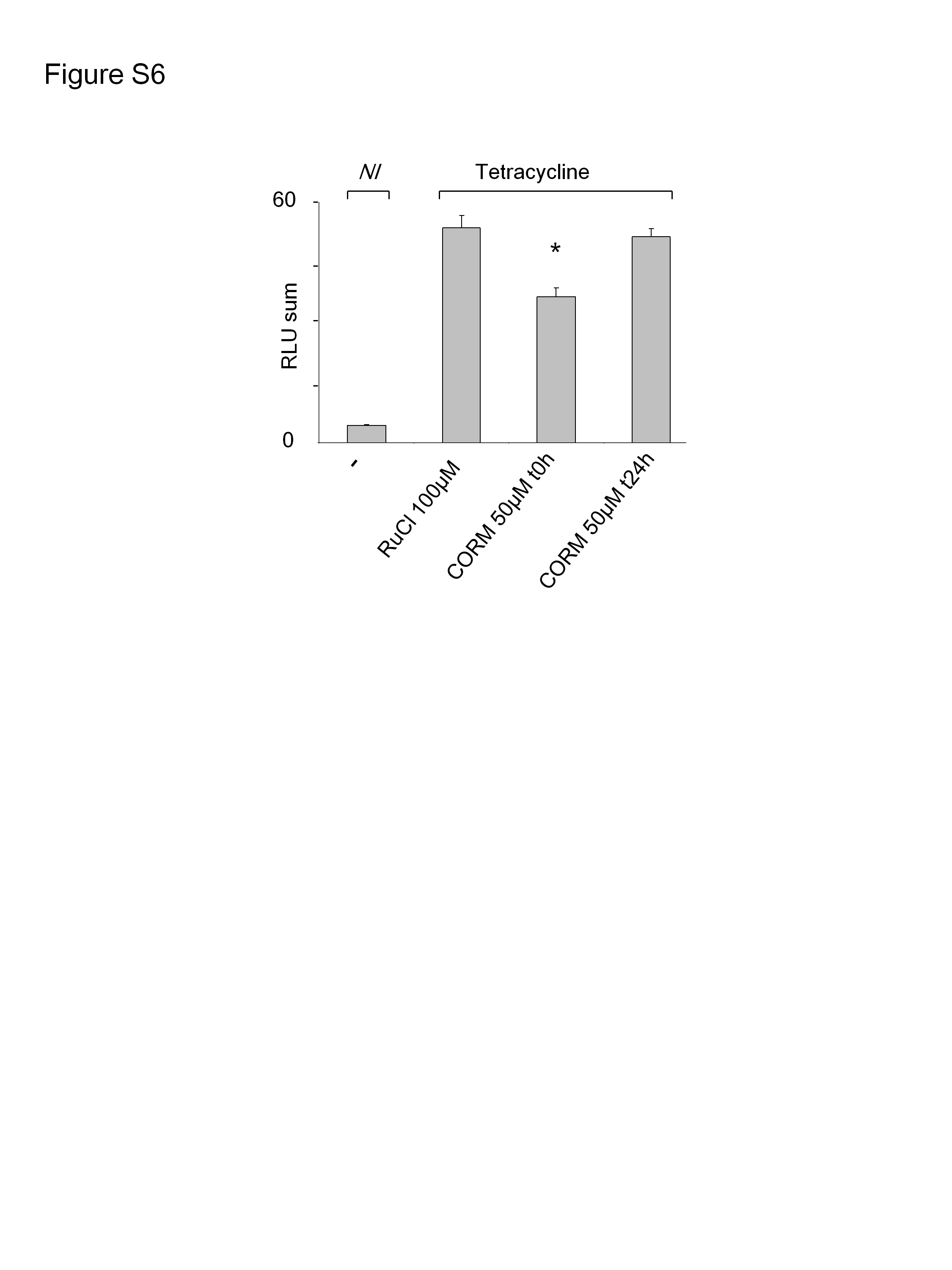

Supplement: Figure S6 — CORM does not exhibit an antioxidant property. The effect of 50 µM CORM extemporaneously prepared (t0h) versus prepared 24h before the experiment (t24h) in which all the CO is volatilized, was investigated on Tet-induced HEK293 T-REx™ cells. Nox4 activity was measured by chemiluminescence. * p<0.05 versus CORM t24h treated cells. Results are representative of three independent experiments. (TIF) [file pone.0066478.s006.tif]
