# Supplementary material for: Heme Oxygenase-1 Regulates Matrix Metalloproteinase MMP-1 Secretion and Chondrocyte Cell Death via Nox4 NADPH Oxidase Activity in Chondrocytes
Source: PLoS One. 2013 Jun 20;8(6):e66478. doi: 10.1371/journal.pone.0066478 (PMC3688771; doi:10.1371/journal.pone.0066478)
Supplement: Text S1 — (DOC) [file pone.0066478.s007.doc]

## Supporting materials and methods:

**Cell viability assay by microscopy**

Nox4A or Nox4B transfected C-20/A4 chondrocytes were cultured in 6 wells plate until they reach confluence in complete DMEM. Medium was then replaced by DMEM depleted for fetal bovine serum and treated or not with 10 ng/ml IL-1β +/- 500 nM N-acetyl cystein. Five days later, the proportion of adherent cells (representative of living cells) and floating cells (representative of dead cells) was illustrated by photography under x100 magnification to acquire a field representative of the cell state.

### Amplex Red assay

HEK293 T-REx™ cells were induced or not for Nox4 expression by tetracycline 1 µg/ml and for HO-1 expression by 10 or 25 µM CoPP-IX. After 48h, cells were detached with 0.05 % (w/v) trypsin, washed twice with PBS and collected after 8 min centrifugation at 400 g at room temperature. The viability of the suspended cells was over 90%, as determined by the trypan blue exclusion method. In a 96-well plate, 5x10^5^ living cells resuspended in 20 µl PBS were added per well. Before the start of the assay, 100 µl of a PBS solution containing 5 µM Amplex Red and 10 mUnits/ml horseradish peroxidase was added in each well. Results are expressed as the sum of Relative Fluorescent Units (RFU) recorded every two minutes during 30 min on a fluostar omega spectrofluorimeter (BMG labtech).

### LDH activity measurement

The activity of LDH was measured in the incubation medium as an index of plasma membrane integrity. C-20/A4 WT cells were induced or not for HO-1 expression by 10 or 25 µM CoPP-IX and treated with 10 ng/ml IL-1β during 5 days with 2% of fetal bovine serum. Supernatants were then collected and assessed for LDH activity on a Hitachi/MODULAR automated analyzer using the Roche optimized kit.
